# Pulmonary Mucormycosis Without Rhinocerebral Involvement: A Case Report

**DOI:** 10.7759/cureus.114992

**Published:** 2026-08-22

**Authors:** Maheen N Mirza, Bryan J Fisher, Jeffrey Schoondyke, Julio D Rodriguez-Velez, Matthew V Tavares

**Affiliations:** 1 Department of Internal Medicine, HCA Florida Citrus Hospital, Inverness, USA; 2 Department of Medicine, Florida State University College of Medicine, Tallahassee, USA

**Keywords:** diabetic ketoacidosis, hyperosmolar hyperglycemic state, immunocompromised state, pneumonia, pulmonary mucormycosis

## Abstract

Mucormycosis is an opportunistic fungal infection that commonly occurs in patients with predisposing conditions, such as poorly controlled diabetes mellitus and immunocompromised states. In patients with diabetes, rhinocerebral disease is more commonly described, whereas isolated pulmonary mucormycosis (PM) is less typical and is more often associated with profound immunocompromise. The presentation in this case was further notable for occurring in the setting of a hyperosmolar hyperglycemic state (HHS) rather than diabetic ketoacidosis (DKA), without rhinocerebral involvement.

We present the case of a 53-year-old man with type 2 diabetes mellitus treated with insulin who initially presented with cough, fatigue, and generalized malaise and re-presented five days later after a syncopal ground-level fall with right shoulder pain and persistent weakness. He was found to have a HHS and sepsis secondary to pneumonia, with sputum and bronchoalveolar lavage cultures growing *Enterobacter cloacae* complex. Imaging obtained during the evaluation of the shoulder injury revealed a nondisplaced proximal humeral fracture and an incidental large cavitary pulmonary lesion, prompting computed tomography of the chest, which demonstrated multiple cavitary consolidations and moderate-to-large bilateral pleural effusions. Bronchoscopy with biopsy demonstrated necrotic tissue containing broad, aseptate fungal hyphae morphologically consistent with mucormycosis. Computed tomography of the sinuses showed no evidence of rhinocerebral involvement. The patient was treated with meropenem and liposomal amphotericin B and was transferred to a tertiary-care facility for surgical evaluation and debridement.

PM should be considered in patients with poorly controlled diabetes who develop pneumonia or cavitary disease, even in the absence of classic rhinocerebral involvement. This case underscores the need for prompt identification of PM, antifungal therapy, and early surgical intervention to improve outcomes.

## Introduction

Mucormycosis is a rare opportunistic fungal infection caused by *Rhizopus* and *Mucor* species. These fungi are found in soil and decaying organic matter and may be introduced through inhalation, ingestion, or direct inoculation through a disrupted skin barrier [1]. Mucormycosis typically occurs in patients with specific predisposing conditions, such as diabetic ketoacidosis (DKA) or immunocompromised states [1]. These fungi tend to invade blood vessels, causing thrombosis, necrosis, and tissue infarction. The diagnosis relies on histologic evaluation demonstrating characteristic broad, irregular, pauciseptate or nonseptate hyphae [1].

Clinical manifestations include rhinocerebral, pulmonary, cutaneous, gastrointestinal, and disseminated disease. Pulmonary mucormycosis (PM) is more commonly described in patients with profound immunocompromise, including those with hematologic malignancies and transplant recipients [2]. Clinical manifestations of PM are nonspecific and may include fever, cough, dyspnea, chest pain, and hemoptysis, while radiographic findings may include consolidation, pleural effusion, nodules or masses, and cavitation [2, 3]. PM is associated with substantial mortality, with an estimated mortality rate of 57.1% [4]. Given the severity of the disease, timely diagnosis, initiation of antifungal therapy, and early surgical evaluation are critical [5].

This case demonstrates the diagnostic challenges associated with recognizing PM in patients with type 2 diabetes presenting with hyperosmolar hyperglycemic state (HHS) rather than DKA, concomitant *Enterobacter cloacae* complex pneumonia, and no evidence of rhinocerebral involvement.

## Case presentation

A 53-year-old man with a past medical history of type 2 diabetes mellitus treated with insulin glargine (35 units nightly) and rapid-acting insulin aspart, end-stage renal disease on hemodialysis, multivessel coronary artery disease with previous coronary artery bypass grafting, heart failure with preserved ejection fraction, chronic obstructive pulmonary disease, and prior admissions for DKA presented to the ED after becoming lightheaded and losing consciousness at home, resulting in a ground-level fall and subsequent right shoulder pain. The patient’s occupation and specific environmental exposures were not documented in the available medical record.

Five days before this presentation, he had presented to the same ED with a one-day history of cough, fatigue, and generalized malaise. Chest imaging at that time demonstrated a left-sided pleural effusion with adjacent airspace disease and additional right-sided pulmonary opacities. He was treated with ceftriaxone for suspected pneumonia and insulin for hyperglycemia but left against medical advice later that day before further microbiologic evaluation or a complete course of antibiotics could be completed.

On re-presentation, he arrived at the ED without EMS involvement and was conscious on arrival. He reported persistent weakness since leaving the hospital and stated that he had not taken his insulin that day, although he reported taking it previously. No additional respiratory complaints were documented at the time of re-presentation. His blood pressure was 150/82 mm Hg, with the remaining initial vital signs documented as stable and within normal ranges. Physical examination was significant for dry oral mucosa and decreased range of motion in the right arm. Laboratory studies were notable for a serum glucose level of 963 mg/dL, corrected sodium of 133 mmol/L, chloride of 84 mmol/L, blood urea nitrogen of 30 mg/dL, creatinine of 5.4 mg/dL with an estimated glomerular filtration rate below 15 mL/min/1.73 m² in the setting of dialysis-dependent end-stage renal disease, anion gap of 15 mmol/L, WBC count of 15.3 × 10³/µL, hemoglobin of 10.1 g/dL, hematocrit of 30.4%, platelet count of 668 × 10³/µL, ESR of 63 mm/hr, and lactate of 3.37 mmol/L. No arterial or venous blood gas results were available in the reviewed record to further characterize the acid-base status. The patient was diagnosed with HHS and started on an intravenous insulin infusion per the department’s protocol. In the setting of pulmonary infection, leukocytosis, elevated lactate, and a subsequent supplemental oxygen requirement of up to 12 L/min, he was also treated for sepsis secondary to pneumonia and started empirically on piperacillin-tazobactam.

Radiographs obtained because of the right shoulder injury were equivocal for fracture, prompting CT of the shoulder. This demonstrated a suspected nondisplaced proximal humeral fracture and incidentally identified a large, thick-walled cavitary lesion in the right upper lobe measuring approximately 8 × 8 × 9 cm, prompting dedicated CT of the chest. Orthopedic surgery recommended conservative treatment of the humeral fracture with immobilization for six weeks. No traumatic wound associated with the fall was documented.

**Figure 1:**
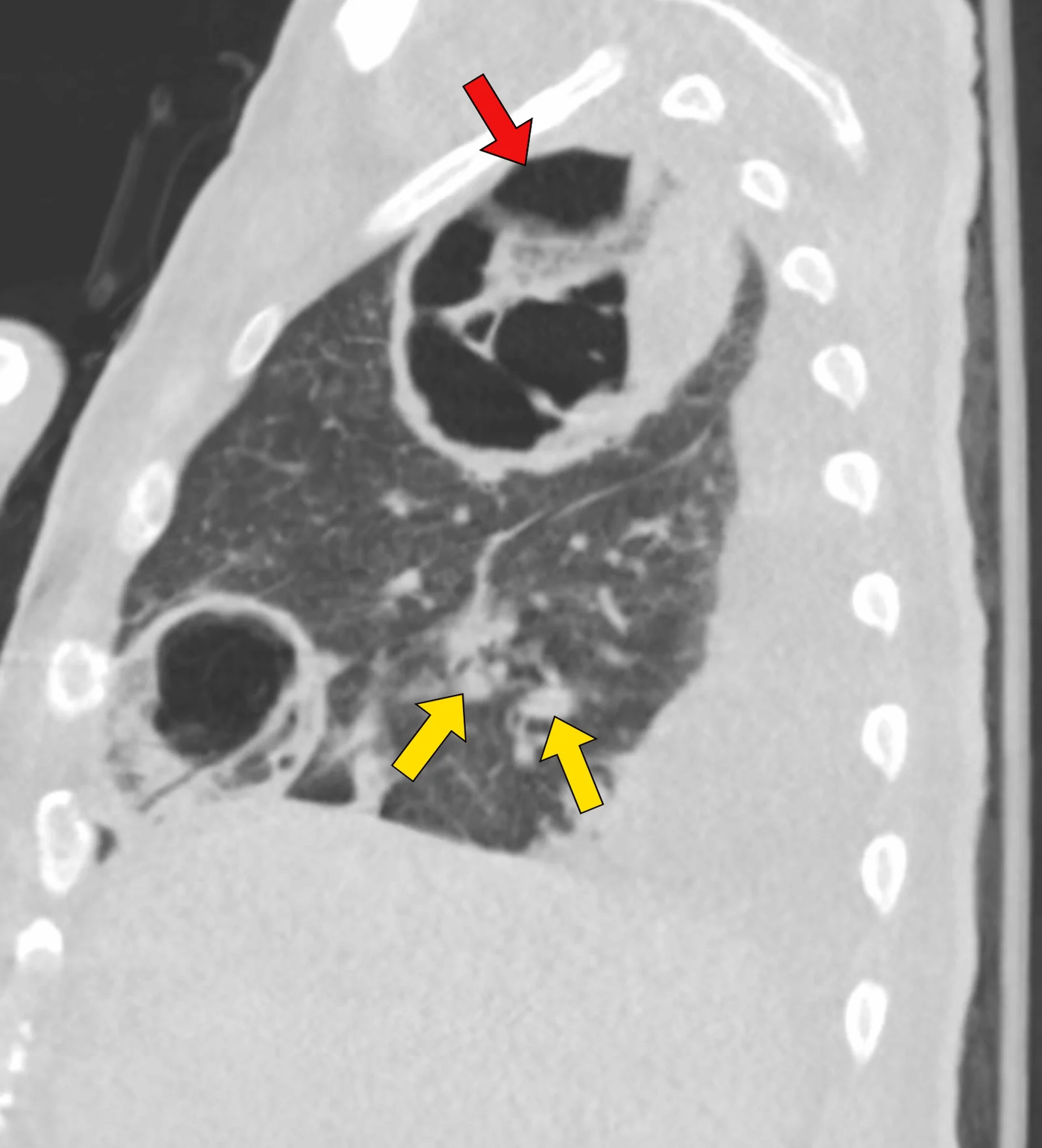
Sagittal CT image of the chest demonstrates the cavitary lesion (red arrow) and pulmonary nodules (yellow arrows). The extensive cavitary disease raised concern for an invasive infectious process in addition to the initially identified bacterial pneumonia.

**Figure 2:**
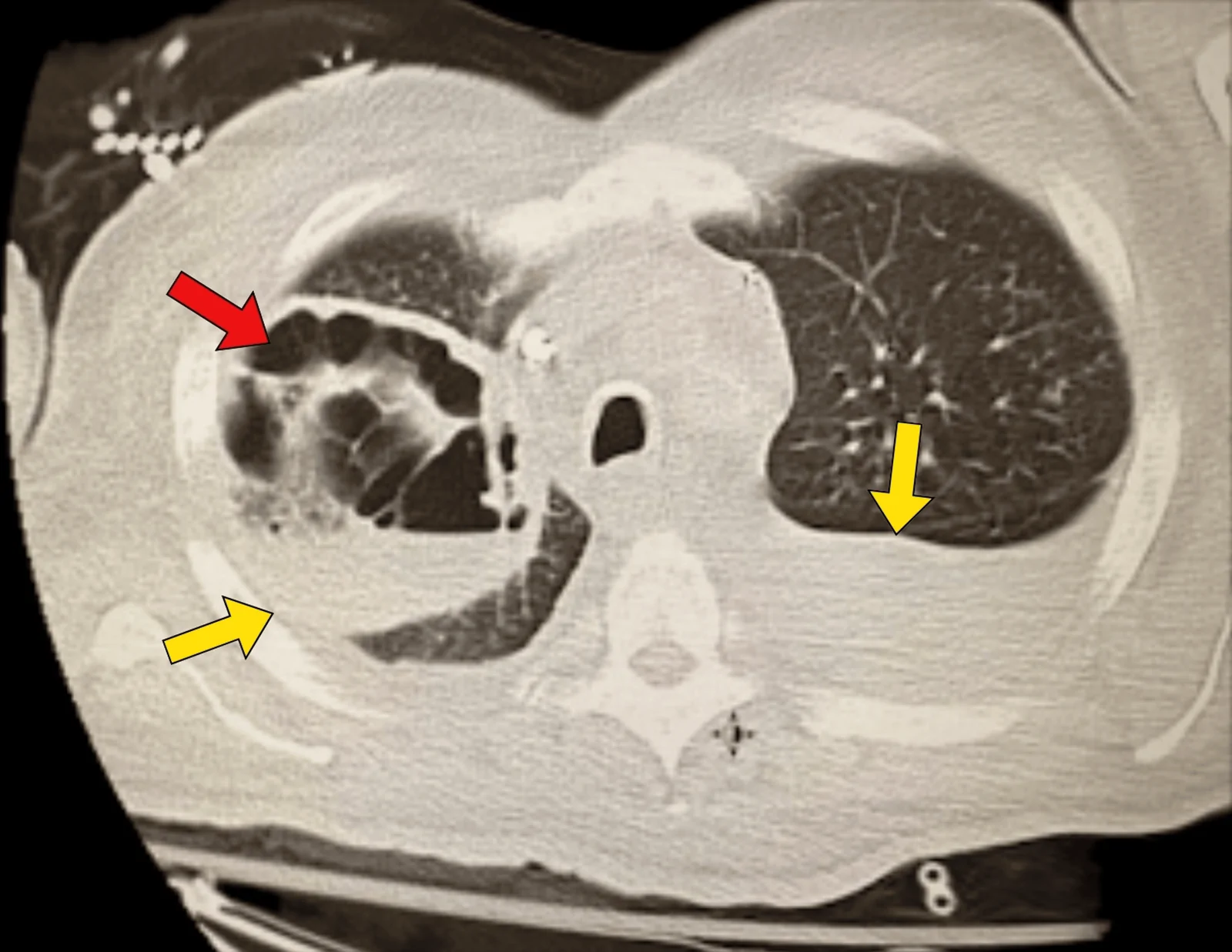
Axial CT image of the chest demonstrates the cavitary lesion (red arrow) and bilateral pleural effusions (yellow arrows). The combination of cavitary disease and pleural effusions prompted further evaluation for an additional infectious or malignant process.

CT of the chest demonstrated a large area of right upper lobe consolidation with cavitary formation, an additional smaller ring-shaped cavitary lesion in the anterior upper lobe, and irregular cavitary consolidation involving the right middle lobe with surrounding pulmonary consolidation. Moderate-to-large bilateral pleural effusions with bibasilar compressive atelectasis were also present (Figures 1-2). Given the multiple cavitary pulmonary lesions, the differential diagnosis included bacterial lung abscess, cavitating pneumonia, and malignancy. Tuberculosis was considered less likely by the treating team because the patient underwent regular medical evaluations for hemodialysis and the pulmonary lesion represented a new radiographic finding. The patient subsequently underwent left-sided thoracentesis with removal of approximately 1,000 mL of pleural fluid and placement of a right-sided pigtail catheter for continued drainage.

**Figure 3:**
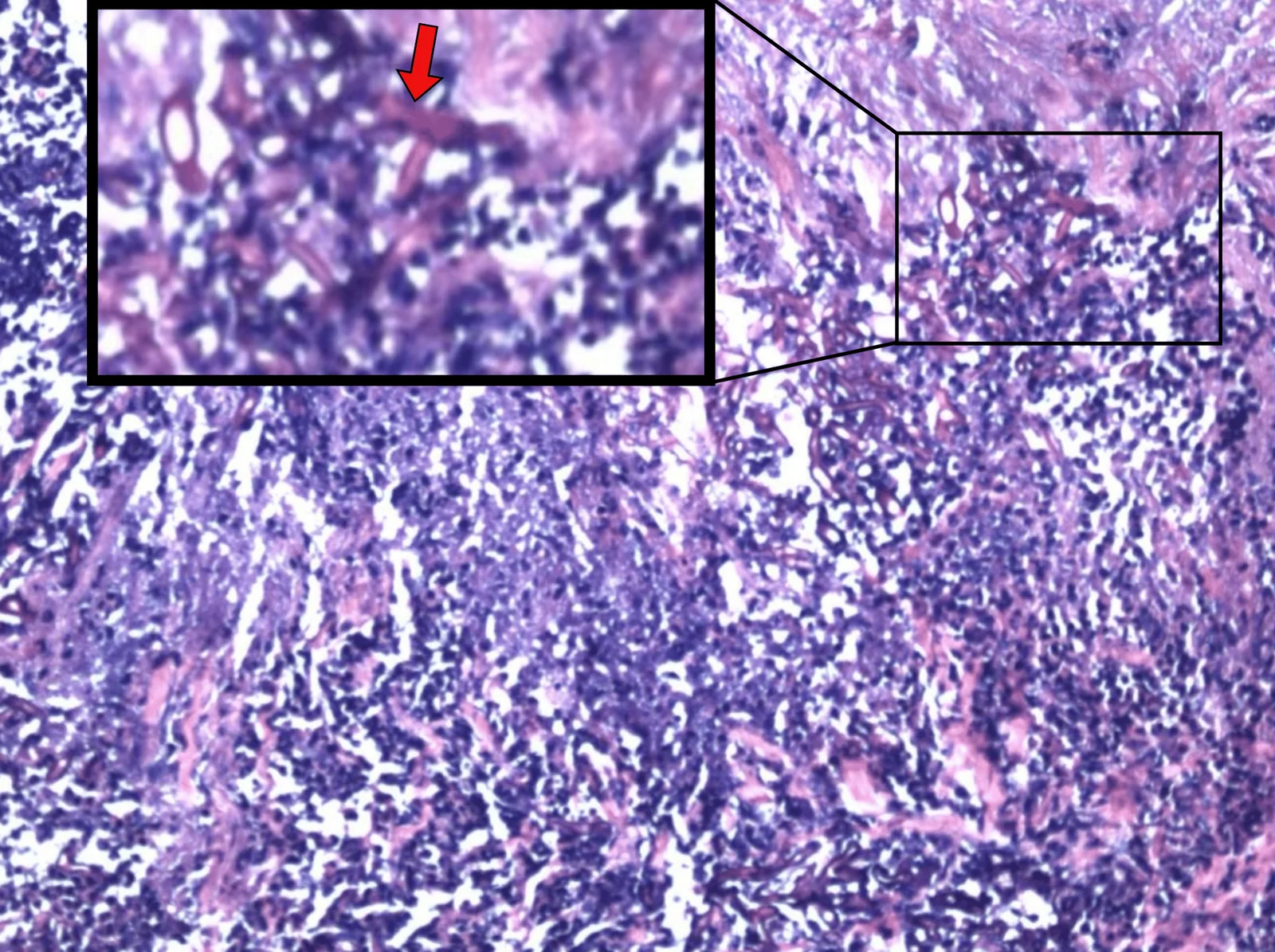
H&E-stained section of the bronchial biopsy showing tissue necrosis with broad, aseptate fungal hyphae morphologically consistent with mucormycosis. Inset: Higher-magnification view highlighting the fungal hyphae (red arrow) identified in the low-power field.

Because the cavitary lesions could not be fully characterized by imaging and malignancy and invasive infection remained in the differential diagnosis, bronchoscopy with washings and a fluoroscopy-guided biopsy was performed. Cultures of sputum and bronchoalveolar lavage fluid grew *Enterobacter cloacae* complex that was resistant to piperacillin-tazobactam, prompting escalation to meropenem. Histopathologic examination of the bronchial biopsy demonstrated fibrotic and necrotic tissue with acute inflammation and fungal organisms morphologically consistent with mucormycosis, without evidence of malignancy (Figure 3). Fungal culture did not yield the causative organism; therefore, species-level identification and antifungal susceptibility testing were not available.

**Figure 4:**
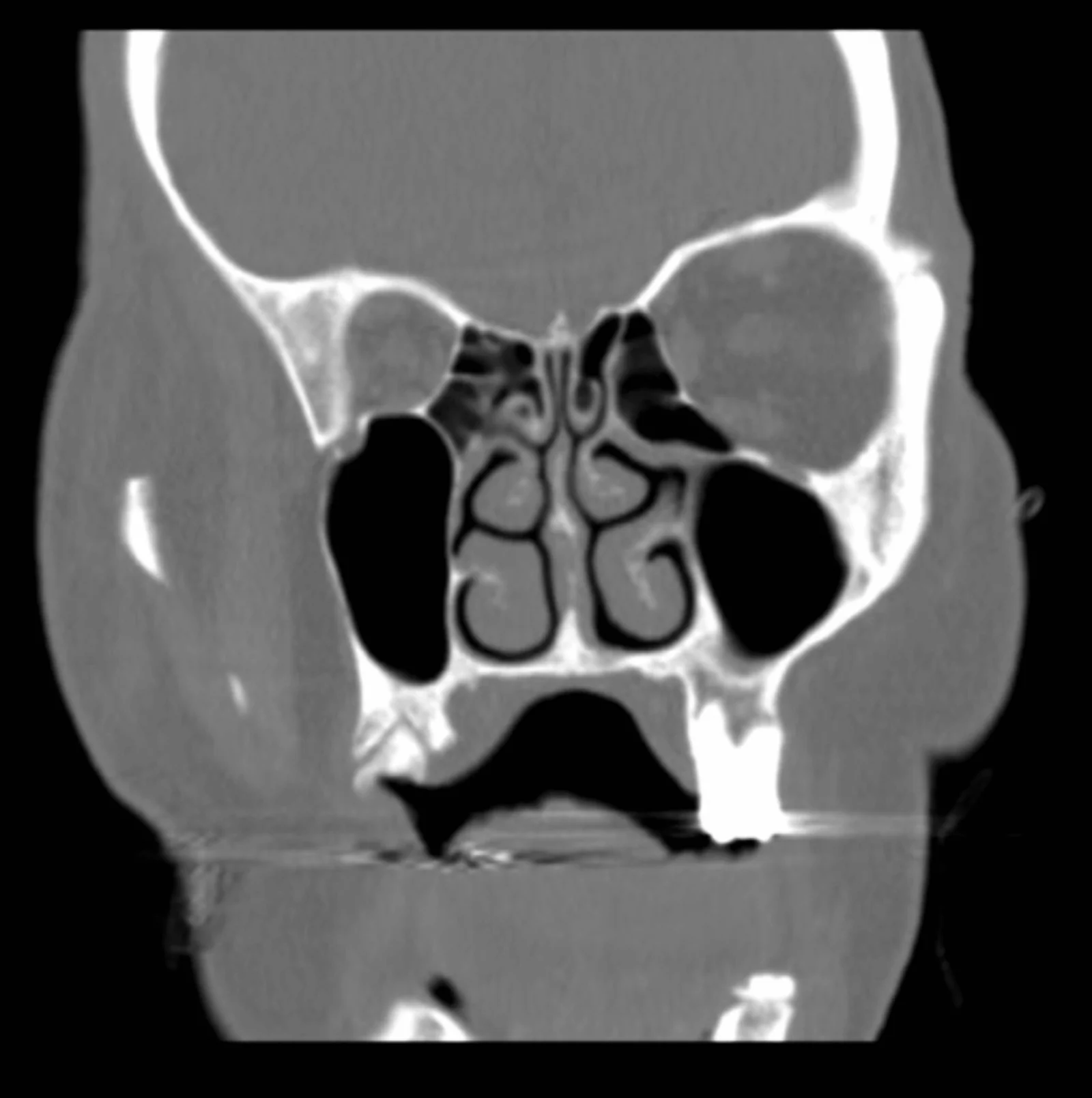
CT of the sinuses showing no evidence of infection. The absence of sinonasal disease supported a pulmonary presentation of mucormycosis without sinonasal involvement.

The patient was started on IV liposomal amphotericin B at 3 mg/kg/day. The selected dose reflected his dialysis-dependent end-stage renal disease with an estimated glomerular filtration rate below 15 mL/min/1.73 m². Cardiothoracic surgery was consulted for surgical management of the necrotic pulmonary disease. CT of the sinuses demonstrated no focal sinonasal disease or evidence of rhinocerebral involvement (Figure 4). He was subsequently transferred to a tertiary-care facility for surgical evaluation and debridement. Records documenting the subsequent operative course, duration of antifungal therapy, or clinical outcome were not available despite review of the accessible medical record.

## Discussion

Mucormycosis in patients with diabetes most commonly manifests with rhinocerebral involvement, whereas pulmonary involvement tends to be more prevalent in immunocompromised patients, such as those with hematologic malignancies or solid organ transplants [6]. Although diabetes is a well-established predisposing condition for the development of mucormycosis, pulmonary involvement in a patient presenting with HHS rather than DKA and without rhinocerebral disease represents a rare presentation. PM is generally acquired through inhalation of environmental Mucorales spores; however, no specific occupational or environmental exposure was identified in this patient.

DKA is associated with mucormycosis in part because of the metabolic disturbances produced by ketoacidosis and hyperglycemia. Ketoacidosis promotes the dissociation of iron from transferrin, increasing available serum iron and creating an environment favorable for fungal growth. Acidosis and hyperglycemia also increase the expression of glucose-regulated protein 78 (GRP78) on endothelial cells, which facilitates fungal adherence and angioinvasion [2]. Additionally, hyperglycemia impairs neutrophil chemotaxis, phagocytosis, and fungal clearance [2]. Diabetes and severe hyperglycemia remain predisposing factors for mucormycosis even in the absence of ketoacidosis. In this case, the patient presented with HHS without documented evidence of ketoacidosis, suggesting that severe hyperglycemia may have contributed to susceptibility despite the absence of the acidosis characteristic of DKA.

In this case, the patient initially presented with cough, fatigue, and generalized malaise, while subsequent imaging demonstrated multiple cavitary pulmonary lesions and bilateral pleural effusions. These findings overlap with bacterial pneumonia, lung abscess, malignancy, tuberculosis, and other invasive fungal infections, underscoring the difficulty of diagnosing PM based on clinical and radiographic findings alone. Concurrent bacterial infection may further complicate the diagnosis of PM because the identification of a bacterial pathogen can provide an alternative explanation for the pulmonary symptoms and radiographic abnormalities. Bacterial co-infection has been reported in patients with PM [7]. In this case, sputum and bronchoalveolar lavage cultures grew *Enterobacter cloacae* complex. Cavitary disease is not a typical radiographic manifestation of *Enterobacter* pneumonia [8].

Histopathologic examination of the bronchial biopsy independently demonstrated fungal organisms morphologically consistent with mucormycosis. The coexistence of these findings emphasizes that the identification of a bacterial respiratory pathogen should not preclude further evaluation of cavitary pulmonary disease when the overall clinical or radiographic picture remains concerning for an additional process. Accordingly, a low threshold for tissue diagnosis should be maintained in at-risk patients with otherwise unexplained cavitary pulmonary lesions.

Management of PM requires prompt antifungal therapy and consideration of surgical intervention when feasible [5]. In this case, liposomal amphotericin B was initiated following histopathologic diagnosis, while the concomitant *E. cloacae* infection was treated with meropenem based on susceptibility testing. The patient was subsequently transferred for surgical evaluation and debridement, although his subsequent clinical course was unavailable.

## Conclusions

This case highlights an atypical presentation of PM in a 53-year-old man with poorly controlled type 2 diabetes who presented with HHS rather than DKA, concomitant bacterial pneumonia, and no evidence of rhinocerebral involvement. Clinicians should maintain a high index of suspicion for invasive fungal infection in patients with diabetes and cavitary pulmonary disease, particularly when the imaging findings are not fully explained by the identified bacterial pathogen. Concurrent bacterial infection should not exclude PM as an additional diagnosis. Early tissue diagnosis, immediate initiation of appropriate antifungal therapy, and surgical evaluation are essential for improving outcomes in this life-threatening infection.
